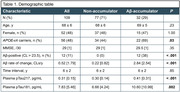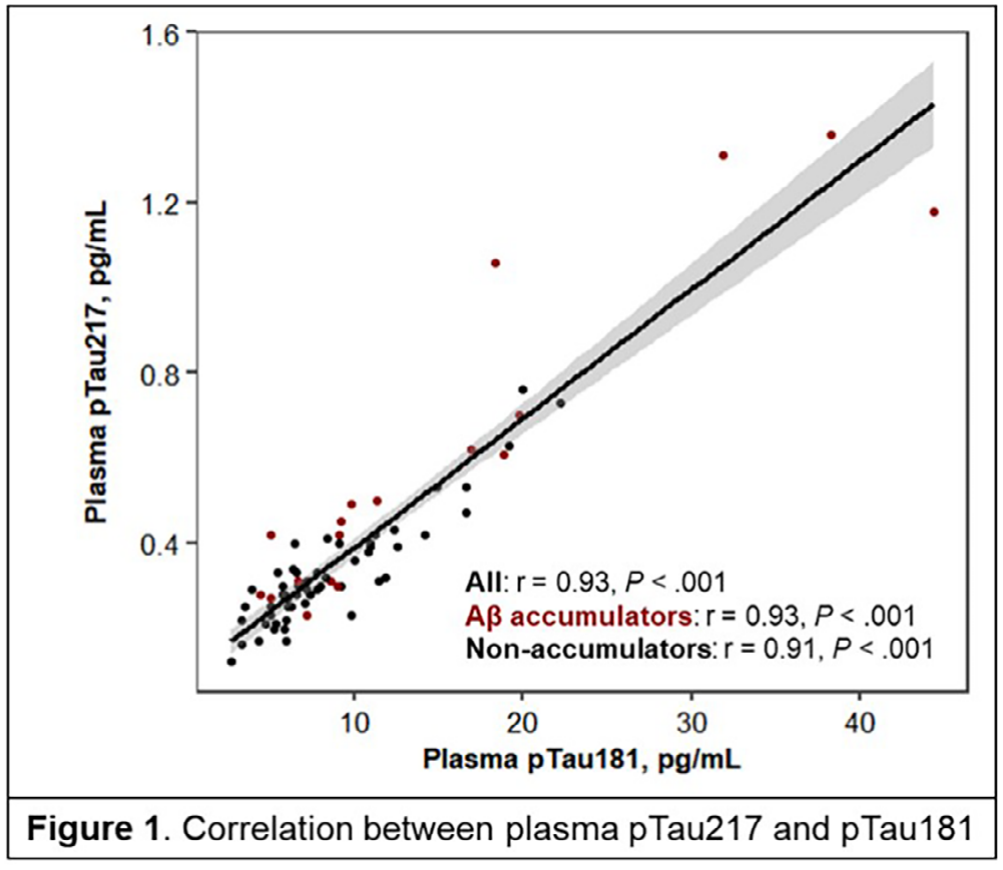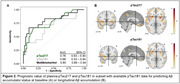# Prognostic value of a novel plasma pTau217 assay for amyloid accumulation in healthy elderly

**DOI:** 10.1002/alz.088710

**Published:** 2025-01-09

**Authors:** Steffi De Meyer, Emma S. Luckett, Jolien Schaeverbeke, Koen Van Laere, Jeroen Vanbrabant, Eugeen Vanmechelen, Patrick Dupont, Guglielmo Di Molfetta, Henrik Zetterberg, Nicholas J. Ashton, Koen Poesen, Rik Vandenberghe

**Affiliations:** ^1^ Laboratory for Molecular Neurobiomarker Research (LaMoN), KU Leuven, Leuven Belgium; ^2^ Alzheimer Research Centre, Leuven Brain Institute, KU Leuven, Leuven Belgium; ^3^ Laboratory for Cognitive Neurology, KU Leuven, Leuven Belgium; ^4^ Karolinska Institutet, Stockholm Sweden; ^5^ KU Leuven, Leuven Belgium; ^6^ University Hospitals Leuven, Leuven Belgium; ^7^ ADx NeuroSciences NV, Ghent Belgium; ^8^ Institute of Neuroscience and Physiology, University of Gothenburg, Mölndal Sweden; ^9^ Department of Psychiatry and Neurochemistry, Institute of Neuroscience and Physiology, the Sahlgrenska Academy at the University of Gothenburg, Mölndal Sweden; ^10^ Department of Psychiatry and Neurochemistry, Institute of Neuroscience and Physiology, The Sahlgrenska Academy, University of Gothenburg, Mölndal, Gothenburg Sweden; ^11^ Laboratory Medicine, UZ Leuven, Leuven Belgium; ^12^ Neurology Department, University Hospitals Leuven (UZ Leuven, Campus Gasthuisberg),, Leuven Belgium

## Abstract

**Background:**

The performance of blood‐based phosphorylated tau (pTau) immunoassays to detect asymptomatic Alzheimer’s disease (AD) has important implications for therapeutic trials. pTau217 is often recommended as the preferred epitope due to its high fold changes in AD. The current study investigates the ability of a novel pTau217 assay to predict the dynamic phase of amyloid‐β (Aβ) accumulation in comparison to the best‐performing pTau181 assay.

**Methods:**

Plasma pTau217 was quantified by the ALZPath Simoa assay at the University of Gothenburg in 109 cognitively unimpaired older adults (Flemish Prevent‐AD Cohort KU Leuven [F‐PACK] cohort). All subjects underwent baseline plasma sampling and longitudinal Aβ‐PET (median time interval = 6 years). For a subset of 72 subjects, pTau181 was quantified by the Homebrew ADx Simoa assay. Linear mixed‐effects models were used to calculate subject‐specific Aβ change and to assess the age‐, sex and APOE‐corrected predictive value of pTau species for Aβ change, which was assessed in a global and voxelwise manner. Accumulators were defined as having an Aβ rate of change z‐score > 1.5 (based on mean & standard deviation within the Aβ‐ subset). Performance to detect Aβ accumulators was assessed through receiver operating characteristic analyses. Plasma biomarkers were converted to z‐scores for effective comparison. Pearson correlations were calculated between pTau181 and pTau217.

**Results:**

Plasma pTau217 levels were higher in Aβ accumulators than non‐accumulators with an area under the curve (AUC) of 0.69 (95%CI 0.58‐0.81). Moreover, higher pTau217 predicted steeper Aβ accumulation (β_s_=0.57, P<.001). Plasma levels of pTau217 strongly correlated with those of pTau181 (r=0.93, Figure 1). In the subgroup with matching pTau181 data, pTau181 was also higher in Aβ accumulators than non‐accumulators with an AUC of 0.68 (95%CI 0.53‐0.84), which was comparable to pTau217 (AUC=0.76; P_DeLong_=.12) or a multibiomarker model including both pTau species (AUC=0.80; P_DeLong_=.15) in the same subset (Figure 2A). pTau181 demonstrated comparable associations with Aβ accumulation in terms of both strength (β_s_=0.53, 95%CI 0.21–0.81 for pTau181 versus β_s_ =0.48 for pTau217 in this subset) and spatial distribution (Figure 2B).

**Conclusions:**

The novel ALZPath pTau217 assay predicted Aβ accumulation in asymptomatic elderly with comparable performance to the ADx pTau181 assay.